# Hyperprogression under Immune Checkpoint Inhibitor: a potential role for germinal immunogenetics

**DOI:** 10.1038/s41598-020-60437-0

**Published:** 2020-02-27

**Authors:** Sadal Refae, Jocelyn Gal, Patrick Brest, Damien Giacchero, Delphine Borchiellini, Nathalie Ebran, Frederic Peyrade, Joël Guigay, Gérard Milano, Esma Saada-Bouzid

**Affiliations:** 1 University Côte d’Azur, Centre Antoine Lacassagne, Oncopharmacology Unit, Nice, France; 2 University Côte d’Azur, Centre Antoine Lacassagne, Epidemiology and Biostatistics Department, Nice, France; 3 University Côte d’Azur, Centre Antoine Lacassagne, CNRS, Inserm, Ircan, FHU-Oncoage, Nice, F-06189 France; 4 University Côte d’Azur, Centre Antoine Lacassagne, Medical Oncology Department, Nice, France

**Keywords:** Predictive markers, Cancer epigenetics

## Abstract

Hyperprogressive disease (HPD), an unexpected acceleration of tumor growth kinetics, is described in cancer patients treated with anti-PD-1/anti-PD-L1 agents. Here, our aim was to take into consideration the host and explore whether single nucleotide polymorphisms (SNPs) in key genes involved in immune response might predispose to HPD. DNA was extracted from blood-samples from 98 patients treated under CPI monotherapy. Four candidate genes (*PD-1*, *PD-L1*, *IDO1* and *VEGFR2*) and 15 potential SNPs were selected. The TGK_R_ (ratio of the slope of tumor growth before treatment and the slope of tumor growth on treatment) was calculated. Hyperprogression was defined as a TGK_R_≥2. TGK_R_ calculation was feasible for 80 patients (82%). HPD was observed for 11 patients (14%) and was associated with shorter overall survival (P = 0.003). In univariate analysis, HPD was significantly associated with age ≥70 y (P = 0.025), immune-related toxicity (P = 0.016), *VEGFR2* rs1870377 A/T or A/A (P = 0.005), *PD-L1* rs2282055 G/T or G/G (P = 0.024) and *PD-L1* rs2227981 G/A or A/A (P = 0.024). Multivariate analysis confirmed the correlation between HPD and age ≥70 y (P = 0.006), *VEGFR2* rs1870377 A/T or A/A (P = 0.007) and *PD-L1* rs2282055 G/T or G/G (P = 0.018). Immunogenetics could become integral predictive factors for CPI-based immunotherapy.

## Introduction

Checkpoint inhibitors (CPIs) including compounds targeting PD-1/PD-L1 axes have brought significant improvements in terms of overall survival in several types of advanced cancers^1–6^. A single response profile, such as pseudo-progression, is observed under CPIs7. Among these typically-related response profiles under CPIs is hyperprogressive disease (HPD) which was defined as an unanticipated and paradoxical acceleration of the tumor growth^7,8^. The incidence of HPD is variable according to the way it is defined and ranges between 4 and 29%^7^. Though such acceleration of the tumor growth kinetic was also observed with other agents (chemotherapy^9^, tyrosine kinase inhibitors^10^), the intensity and the frequency of the phenomenon appears to be higher with checkpoint inhibitors used alone^7^. A single response profile, such as pseudo-progression, is observed under CPIs^7^. Among these typically-related response prfiles under CPIs is hyperprogressive disease (HPD), which has been defined as an unanticipated and paradoxical acceleration of tumor growth^7,8^. The incidence of HPD is variable according to the way it is defined and ranges between 4 to 29%^7^. Although this acceleration of tumor growth kinetics was also observed with other agents (chemotherapy^9^, tyrosine kinase inhibitors^10^), the intensity and frequency of the phenomenon appears to be higher with checkpoint inhibitors used alone^7^. HPD may be associated with a worsening of the outcome^11^. Different physiopathological hypotheses have been tested to explain phenomena such as tumoral genomics variations^12,13^. Indeed, CPI has been shown to hasten tumor growth in a mouse model with a relative lack of PD-1 expression^14^. As HPD was observed in several malignant tumor types, a role for the host variations has been advocated^13,15,16^. Indeed, allelic variations of HLA class I genes have been shown to impact clinical outcome under CPI^17^. However, dedicated germinal immunogenetics studies remains rare in the context of CPI-based treatment^18^. To better elucidate the potential relationship between host immunogenetics and CPI treatment outcome and particularly HPD, we correlated the outcome of patients treated with CPI and selected polymorphisms described in four key genes: *PD-1* (Programmed Cell Death 1 gene, 2q37.3), *PD-L1* (Programmed Death Ligand 1 gene, 9p24.1), *IDO1* (Indoleamine 2,3-Dioxygenase 1 gene, 8p11.21) and *VEGFR2 (Vascular Endothelial Growth Factor Receptor 2* gene, 4q12).

## Results

### Patient characteristics and outcome

Patient baseline characteristics are given in Table 1. All patients were treated for an advanced malignancy. Non-small cell lung cancer (NSCLC) (n = 48) was the largest subgroup followed mainly by head and neck squamous cell carcinoma (HNSCC) (n = 16), renal cell carcinoma (RCC) (n = 14) and melanoma (n = 13). Importantly, all patients were treated by CPI monotherapy alone (anti-PD-1 or anti-PD-L1), with a majority of anti-PD1 (87%). Median age was 68 (range: 32–85), 65 were males (66%) and 70 were smokers (83%). Sixty-six patients had received previous irradiation (69%). The SNP genotype, gene information and genotype frequency are shown in Table 2.

**Table 1:** Patient characteristics.

Variable	No of patients	%
Median Age_(min-max)_	68_32–85_	
**Gender**		
Female	33	34
Male	65	66
**Histology**		
Non-small cell lung cancer	48	49
Head and neck squamous cell	16	16
Carcinoma Melanoma	13	13.5
Renal cell carcinoma	14	14.5
Others (2 bladder, 2 ovarian, 2 hematological, 1 gastrointestinal)	7	7
**Smoker**		
No	14	17
Yes	70	83
**Previous irradiation**		
No	30	32
Yes	66	68
N/A	2	
**Number of lines before recurrence**		
0	15	15.5
1	53	54
2	20	20.5
3	6	6
≥4	4	4
**Anti-PD-1/PD-L1**		
Anti-PD-1	85	84
Anti-PD-L1	13	14
**Reason for stopping treatment**		
Progression	33	75
Toxicity	6	14
Prolonged response	4	9
Patient	1	2
N/A	54	
**Response**		
Complete response	8	8
Partial response	43	44
Stable disease	28	28.5
Progressive disease	19	19.5
**irAE**		
0	16	16.25
1–2	67	68.25
3–4	15	15.5
**Type IrAE**		
Hematologic	18	20
Dermatologic	18	20
Thyroid	13	14.5
Digestive	7	7.5
Metabolic	5	5.5
Articular	12	13.5
Rhinitis	5	5.5
Others	12	13.5
**TGK**_**R**_		
<2	69	86
≥2	11	14
N/A	18	

Abbreviations: N/A = Not Available; Anti PD-L1 = Anti-programmed cell death ligand1; Anti PD-1 = Anti-programmed cell death; TGKR= Tumor growth kinetic rate.

**Table 2:** Summary of genotyping results by MassARRAY (AGENA) of 98 patients.

Gene SNPs	PD-1			PD-L1					VEGFR2			IDO1			
	rs10204525	rs11568821	rs2227981	rs2282055	rs2297136	rs2297137	rs4143815	rs10815225	rs2305948	rs1870377	rs2071559	rs3739319	rs3808606	rs373931	rs9657182
Population	C/C (81)	C/C (74)	A/A (12)	T/T (49)	G/G (16)	G/G (52)	G/G (42)	G/G (68)	C/C (84)	T/T (63)	A/A (25)	G/G (23)	A/A (12)	T/T (3)	C/C (17)
	C/T (17)	C/T (21)	A/G (42)	G/G(3)	A/A (28)	A/A (4)	C/C (13)	C/C (4)	C/T (14)	A/A (3)	A/G (23)	A/A (25)	A/G (57)	C/C (65)	C/T (53)
	T/T (0)	T/T (1)	G/G (41)	G/T (46)	A/G (54)	A/G (41)	C/G (42)	C/G (24)	T/T (0)	A/T (32)	G/G (50)	A/G (50)	G/G (29)	C/T (30)	T/T (28)
Ancestral allele	C/T Ancestral:A	C/T Ancestral:C	A/G Ancestral:G	T/G Ancestral:T	G/A Ancestral:G	G/A Ancestral:G	G/C Ancestral:G	G/A Ancestral:G	C/T Ancestral:C	T/A Ancestral:T	A/G Ancestral:A	G/A Ancestral:G	A/G Ancestral:G	T/C Ancestral:C	C/T Ancestral:T
Minor allele frequency	0.35 (T)	0.04 (T)	0.35 (A)	0.30 (G)	0.33 (G)	0.23 (A)	0.28 (C)	0.16 (C)	0.15 (T)	0.21 (A)	0.5 (A)	0.41 (A)	0.46 (A)	0.16 (T)	0.45 (C)
SNPs Functional Impact	3′UTR variant	Intron variant	Synonymous variant	Intron variant	3′UTR variant	Non-coding transcript exon variant	3′UTR variant	Upstream gene variant	Missense variant	Missense variant	Upstream gene variant	Intron variant	Intron variant	Intergenic variant	Intron variant

Median follow-up was 13.3 months (95% confidence interval [CI]; 10.6 months to 15.4 months). Median irPFS was 16.8 months (95% confidence interval [CI]; 10.2 months to NA) and median OS was not reached. Twelve-month OS and 12-month PFS were 80% (95% confidence interval [CI], 72% to 90%) and 47% (95% confidence interval [CI]; 5% to 60%), respectively.

**Figure 1:**
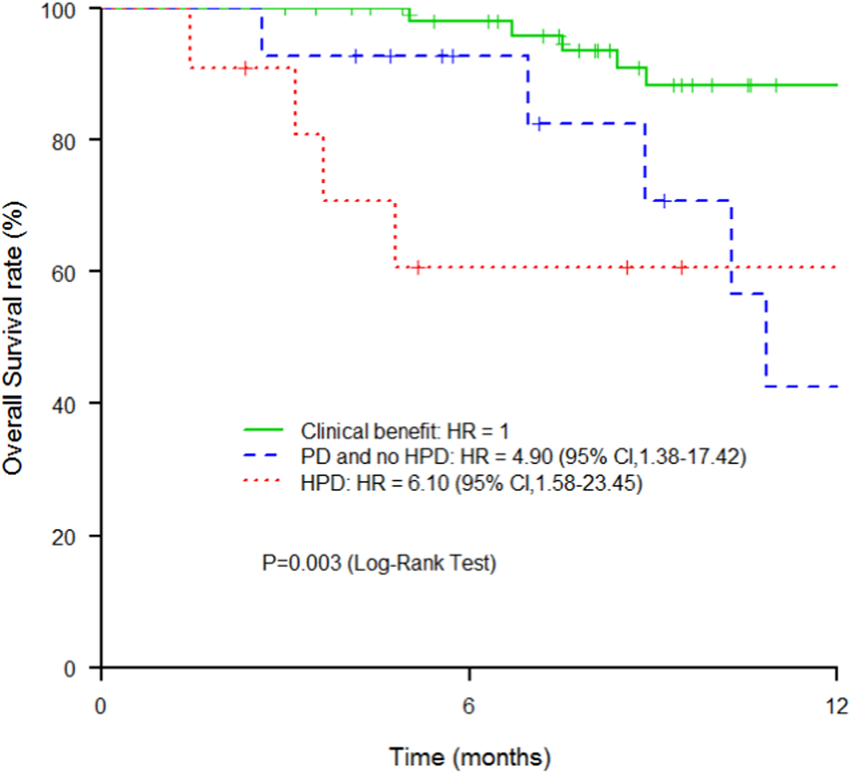
Association between HPD and OS: Kaplan Meier estimates of OS of patients treated with anti PD1/anti PDL1 according to ir-RECIST criteria: clinical benefit (complete response, partial response, stable disease), PD non HPD (progressive disease) and HPD.

Fifteen patients experienced grade 3–4 IrAEs (15.5%), 67 grade 1–2 IrAEs (68.25%) and 16 patients had no IrAE (16.25%). Overall response was complete for 8 patients (8%), partial for 43 patients (44%), stable disease for 28 patients (28.5%) and progressive disease for 19 patients (19.5%). TGK_R_ could be calculated for 80 patients (15 patients had CPI as first line for advanced disease; pre-baseline scanner was not available for 3 patients). HPD was observed in 11 patients (14%). HPD was correlated with shorter OS (Fig. 1) compared with non-HPD patients (P = 0.003).

### HPD predictive factors

In univariate analysis (Table 3), HPD was significantly associated with age ≥70 years (25% versus 6%; P = 0.025), immune-related toxicity grade ≥3 (38.5% versus 9.5%; P = 0.016), *VEGFR2* rs1870377 A/T or A/A (26% versus 4%; P = 0.005), *PD-L1* rs2282055 G/T or G/G (23% versus 2.5%; P = 0.024) and *PD-L1* rs2227981 G/G (4.5% versus 23.5%; P = 0.024). HPD was not significantly correlated with lactate dehydrogenase (LDH) blood levels at baseline (p = 0.055). Similarly, the neutrophil-to-lymphocyte ratio (NLR) was not linked to HPD (p = 0.936). Also, tumor burden was not associated with HPD (p = 0.732). Multivariate analysis revealed an independen t association between HPD and age ≥ 70 years (OR = 14.42; 95% confidence interval [CI];  2 to 100; P = 0.006), rs1870377 T/A or A/A, and *VEGFR2* (OR = 15.36; 95% confidence interval [CI]; 1.92 to 119; P = 0.007) and rs2282055 T/G or G/G, *PDL1* (OR = 17.73; 95% confidence interval [CI]; 11.55 to 227; P = 0.01).

**Table 3:** Univariate and multivariate analyses for hyperprogressive disease.

Parameters	Univariate Analysis					Multivariate Analysis						
						Initial Model^a^			Final Model^b^			P value
	TGK_R<2_ (N = 69)	TGK_R≥2_ (N = 11)	OR	95% CI	P value ^h^	Estimate	SE	P value	Estimate	SE	OR [95% CI]	
**Age (year old)**												
<70	45 (94)	3 (6)	1	reference		reference			Reference		1	
≥70	24 (75)	8 (25)	5	[1.21–20.61]	0.025	2.17	1.28	0.09	2.66	0.97	14.42 [2–100]	0.006
**Gender**												
Male	19 (76)	6 (24)	1	reference		—	—	—	—	—	—	—
Female	50 (91)	5 (9)	0.31	[0.08–1.16]	0.089	—	—	—	—	—	—	—
**Histology**												
Non-small cell lung cancer	14 (93.5)	1 (6.5)	—	—		—	—	—	—	—	—	—
Head and neck squamous cell	38 (86.5)	6 (13.5)	—	—		—	—	—	—	—	—	—
Carcinoma Melanoma	4 (100)	0 (0)	—	—		—	—	—	—	—	—	—
Renal cell carcinoma	11 (91.5)	1 (8.5)	—	—		—	—	—	—	—	—	—
Others^f^	2 (40)	3 (60)	—	—	0.078	—	—	—	—	—	—	—
**Smoker**												
No	9 (100)	0 (0)	1	reference		—	—	—	—	—	—	—
Yes	51 (85)	9 (15)	1.17 ^g^	[1.05–30]	0.594	—	—	—	—	—	—	—
**Previous irradiation**^**i**^												
No	17 (85)	3 (15)	1	reference		—	—	—	—	—	—	—
Yes	51 (86.5)	8 (13.5)	0.88	[0.21–3.73]	1	—	—	—	—	—	—	—
**Number of lines before recurrence**												
0	5 (100)	0 (0)	—	—		—	—	—	—	—	—	—
1–4	64 (85)	11 (15)	—	—	1	—	—	—	—	—	—	—
**Anti-PD-1/PD-L1**												
Anti-PD-1	59 (87)	9 (13)	1	reference		—	—	—	—	—	—	—
Anti-PD-L1	10 (83)	2 (17)	1.3	[0.24–6.9]	0.667	—	—	—	—	—	—	—
**Immune related Adverse Event**^**d**^												
<3	47 (90.5)	5 (9.5)	1	reference		reference			—	—	—	NS ^c^
≥3	8 (61.5)	5 (38.5)	5.87	[1.38–25.01]	0.016	1.71	1.14	0.13	—	—	—	—
**Lactate dehydrogenase (LDH, UI/L)**^**j**^												
	338.5 (109–1269)	414 (252–770)			0.055	—	—	—	—	—	—	—
NLR^k^	3.6 (0.72–63.52)	2.6 (2.64–37)			0.936							
Tumor burden^l^	57 (12–189)	59 (10–143)			0.732							
***VEGFR2 rs1870377***												
*T/T*	46 (96)	2 (4)	1	reference		reference			Reference		1	
A/T or A/A	23 (74)	8 (26)	9	[1.79–45.1]	0.005	3.98	1.69	0.018	2.73	1.02	15.36 [1.92–119]	0.007
***PD-L1 rs2282055***												
*T/T*	36 (97.5)	1 (2.5)	1	reference		reference			Reference		1	
*G/T or G/G*	33 (77)	10 (23)	10.90	[1.32–89.90]	0.024	2.93	1.59	0.06	2.93	1.24	17.73 [1.55–227]	0.018
***PD-L1 rs2227981***^***e***^												
*G/A or A/A*	26 (76.5)	8 (23.5)	1	reference		reference			—	—	—	NS^c^
*G/G*	41 (95.5)	2 (4.5)	6.30	[1.24–32.05]	0.024	1.83	1.30	0.15	—	—	—	—

Significant p values are bolded; ^a^Initial model: including all variables with P < 0.05 in univariate analysis; ^b^Final model: same model after backward stepwise algorithm; ^c^NS = not significant after stepwise algorithm; ^d^Data available for 65 patients; ^e^Data available for 77 patients; ^f^2 bladder, 2 ovarian, 1 gastrointestinal; ^g^Relative Risk [95% CI]; ^h^Fisher’s exact or Wilcoxon’s test; ^i^Data available for 79 patients; ^j^median (min-max), Baseline data available for 55 patients: N = 48 for TGKR <2 and N = 7 for TGKR ≥2; ^k^Neutrophil-to Lymphocyte Ratio; median (min-max); ^l^Sum of the largest diameter of target lesions at baseline, median (min-max).

A risk score was calculated by logistic regression and integrated the 3 independent variables (age, rs2282055, rs1870377*)* for predicting HPD. The risk for HPD was optimally estimated (OR = 18.34; 95% confidence interval [CI]; 3.38 to 99.58; P <0.001) (Table 4).

**Table 4:** Classification of patients based on risk group and risk evaluation of each group.

Risk group	Total n (%)	Hyperprogressive disease		Odds Ratio (CI 95%)	p
		No HPD	HPD		
Low risk	69 (86.25%)	66 (95.5%)	3(4.5%)	referent	
High risk	11 (13.75%)	6 (54.5%)	5 (45.5%)	18.34 [3.38–99.58]	<0.001

## Discussion

We observed HPD in 14% of treated patients by CPI, a figure in the range of figures reported in independent series^7^. We identified older age as a predictive variable for HPD in accord with previously reported series^11^. However, this point is controversial and observations have been reported in recent studies by Kim *et al*.^19^ and Ferrara *et al*.^9^ showingno association between HPD and age. These discrepancies may be due to the different evaluation methods used to evaluate HPD as well as to the retrospective nature of these studies. In agreement with others^19^, we noted that patients with HPD had higher baseline LDH levels but which did not reach statistical significance in our hands. Our negative finding contrasts with that of Kim and coworkers^19^ reporting that patients with HPD had baseline NLR values higher than those of patients without HPD. This discrepancy can be explained by the retrospective nature of both studies and also by the relatively small number of patients. Clearly, prospective studies based on a larger set of patients would be more likely to provide firmer conclusions regard of this possible association between baseline NLR and the risk to developing HPD under CPI. To the best of our knowledge, the present study is the first cohort that explores the link between host gene polymorphisms and HPD under CPI. Our data highlight two germinal variations with rs2282055 (*PD-L1*) and rs1870377 (*VEGFR2*) having a significant and independent influence on the occurrence of HPD.

The group of patients with rs2282055 (*PD-L1*) G allele, either homozygous or heterozygous, was found to be significantly associated with a higher risk of developing HPD in comparison with T/T genotype, the locus being located on chromosome 9p24.1. When expressed on tumor cells, this gene down-regulates the activation of T effector cells through a key mechanism responsible for immune response evasion^20^. However, the real impact of tumor *PD-L1* expression on treatment outcome under CPI remains controversial^21^. The regulation of tumoral and non-tumoral *PD-L1* expression is a complex phenomenon and is influenced by multiple molecular pathways^22–24^. rs2282055 (*PD-L1*) is associated with 10 other SNP all inserted in different introns of the *PD-L1* gene^25^. It has been shown that introns may have a direct or indirect influence on mRNA expression: GTEX portal (https://gtexportal.org/home/) indicates that rs2282055 is associated with down-regulated expression of *PD-L1* (*CD274* gene) in brain tissue while it is overexpressed in the pancreas, suggesting that rs2282055 may impact *PD-L1* expression differently in different tissues. rs2282055 (*PD-L1*) was recently evaluated for its association with survival of patients not treated by CPI^26^. In this latter study, the impact of rs2282055 (*PD-L1*) polymorphism on survival was found to be non-significant, thus suggesting a non-prognostic role of this polymorphism. Since *PD-L1* expression was not available in our cohort, we could not examine potential links between this rs and the level of expression of *PD-L1* protein In conclusion, it can be suggested that rs2282055 (*PD-L1*) may interfere with CPI-HPD development, while the underlying mechanism remains to be elucidated.

*VEGFR2* is a gene encoding for vascular endothelial growth factor receptor 2 expressed on both endothelial cells and various immune cells^27,28^. *VEGFR2* is a key regulator of tumor angiogenesis and tumor microenvironment by mainly promoting a high level of Tregs and by reducing the ability of T effector cells to penetrate the tumor cell bed^29^. Of note, rs1870377 (*KDR*, *VEGFR2*, NM_002253.3:c.1416A>T) induces a missense substitution Q472H in the fifth (out of seven) extracellular Ig-like motifs that has been shown to increase VEGF-A binding and activity inducing increased microvessel density in tumor tissue of patients with non-small cell lung cancer^30^. In our series, carriers of rs1870377 (*VEGFR2*) with any A genotype were more prone to develop HPD. Thus, *VEGFR2* substitution Q472H may play a potential role in increased tumor size due to increased angiogenesis and microvessel development in these patients. It is thus conceivable that the impact of *VEGFR2* on tumor and its microenvironment may differ according to the allelic inheritance of the host with an influence on HPD development under CPI.

Collectively, one can formulate a working hypothesis with HPD occurring in a subset of patients harboring unfavorable alleles which modulate the expression of different genes inducing tumor progression under CPI. It was interesting to identify key immunology-linked genes like *PD-L1* and *VEGFR2* gene variants using this approach. The present reported results remain challenging in clinical practice with particular attention given to the fact that most allelic variations are present at relatively low frequencies. However, this study contains a number of limitations which do not allow drawing definitive conclusion: the sample size is relatively small (11 HPD cases) and patients received two different classes of *PD-1* and *PD-L1* CPI. TGK_R_ was not assessable for first-line treated patients. The study covered different histological types and some patients had been more or less heavily pretreated. According to the meta-analysis by Kim and coworkers^31^, the histological type of the tumor is not predictive value for the occurrence of HPD. However, it has been reported that renal cell carcinoma (RCC) patients may be at a lesser risk of HPD^11,32^. Of note, our cohort was also enriched with long-responding patients as all patients alive and treated with CPI in the department were asked their consent to dedicated blood sampling for the study. This explains the high response rate reported in our series (52%). Above all, the study remains original leading to identification of potential host-linked biomarkers for HPD prediction. Interestingly, it was possible to establish a powerful (OR = 18.34; 95% confidence interval [CI]; 3.38 to 99.58; P <0.001) predictive score combining host characteristics such as age and germinal gene polymorphisms. Evaluating the risk of HPD by testing host immunogenetics must remain probabilistic in nature and may differ according to ethnic population, thus limiting extrapolation of the present study outside the Caucasian population. Efforts to expand other candidate genes and their polymorphisms are currently ongoing in larger prospective cohorts. Particular attention should be paid to allelic variations of HLA class I genes.

Finally, our results support the notion of a genetic susceptibility potentially impacting the development of HPD in a Caucasian population. In a broader perspective, it is hoped that the present data can stimulate further studies integrating both somatic and germinal variability aimed at satisfying the still unmet need for faithful predictive biomarkers to ensure enhanced management of cancer therapy by CPI.

## Patients and Methods

### Study design and patients

This is a retrospective study covering the period April to August 2018. All data were retrieved from the clinical database of the Centre Antoine Lacassagne (Nice, France). Tumor responses were evaluated after monotherapy according to RECIST 1.1 criteria (complete response (CR), partial response (PR), stable disease (SD), and progressive disease (PD)). Objective response was evaluated as previously published^33–35^. Immune-related adverse events (irAEs) were evaluated according to National Cancer Institute Common Terminology Criteria for Adverse Events (NCI-CTCAE V5). Pre-baseline, baseline, and initial imaging results were recorded and were to calculate the TGK_R_ (ratio of the slope of tumor growth before treatment and the slope of tumor growth during treatment), as previously reported^8^. The sum of the largest diameter of target lesions at baseline indicated the tumor burden at baseline. HPD was defined as a TGK_R_ ≥2. Written informed consent was systemically obtained before collecting a study-dedicated blood sample. Patient characteristics, at baseline, also included age, gender, histology, smoker status, lactate dehydrogenase (LDH), neutrophil-to-lymphocyte (NLR) and tumor burden.

### SNP selection and genotyping

Seventeen SNPs of *PD-1* (rs10204525; rs11568821; rs22727981), *PD-L1* (rs2282055; rs2297136; rs2297137; rs4143815; rs10815225; rs822339), *IDO1* (rs3739319; rs3808606; rs373931; rs9657182; rs34820341) and *VEGFR2* (rs2305948; rs1870377; rs2071559) were selected according to their functional and/or clinical relevance. Genomic DNA was extracted from a blood sample using the commercially-available Maxwell^®^ 16 LEV Blood DNA Kit (#AS1290, Promega). The assay to screen the 17 SNPs was created by using Assay Design Suite v2.0 (AGENA Bioscience online software) with the “Genotyping Design”option. We had created the assay to screen the 17 SNPs. Data were verified and compatible with DNA controls polymorphism for 15 SNPs; the remaining 2 SNPs had been eliminated (*PD-L1* rs822339 and *IDO1* rs34820341) because incompatible with DNA control polymorphism (https://www.coriell.org/1/NIGMS/Collections/CEPH-Resources). For 15 SNPs minor allele frequency was ≥5% in Caucasians according to SNPpedia (http://www.snppedia.com) and the Ensemble database (http://www.Ensembl.org). All tested SNPs were in Hardy-Weinberg equilibrium (Table 2).

### Statistical considerations

The link between the 15 SNPs and clinico-radiological parameters and CPI response according to ir-RECIST^35^ criteria and irAEs was examined. Statistical comparisons were performed using χ^2^ test or Fisher’s exact test for categorical data and Student’s test or Wilcoxon’s test for continuous variables. Immune-related progression-free survival (irPFS) and Overall Survival (OS) were respectively calculated from the baseline CT scan to progression (according to ir-RECIST criteria) or death and presented graphically using the Kaplan-Meier method. All variables significant at the 5% level in both univariate and multivariate logistic regression models were included. Co-linearity between all variables of the initial multivariate model was evaluated. The choice of the final model was made by performing a backward stepwise selection model. A fitted score for each participant by logistic regression was used to define two risk groups of patients (low or high risk of HPD). The optimal number of risk groups for predictive models was obtained using the Younden method^36^. Statistical analyses were performed using R version 3.5.0 on Windows®.

### Ethical approval

All procedures performed in studies involving human participants were in accordance with the ethical standards of the institutional and/or national research committee (French National Commission for Informatics and Liberties N°17010).

### Informed consent

All patients provided written informed consent before enrollment.
