# Author Correction: Hyperprogression under Immune Checkpoint Inhibitor: a potential role for germinal immunogenetics

**DOI:** 10.1038/s41598-020-66841-w

**Published:** 2020-06-12

**Authors:** Sadal Refae, Jocelyn Gal, Patrick Brest, Damien Giacchero, Delphine Borchiellini, Nathalie Ebran, Frederic Peyrade, Joël Guigay, Gérard Milano, Esma Saada-Bouzid

**Affiliations:** 1 University Côte d’Azur, Centre Antoine Lacassagne, Oncopharmacology Unit, Nice, France; 2 University Côte d’Azur, Centre Antoine Lacassagne, Epidemiology and Biostatistics Department, Nice, France; 3 University Côte d’Azur, Centre Antoine Lacassagne, CNRS, Inserm, Ircan, FHU-Oncoage, Nice, F-06189 France; 4 University Côte d’Azur, Centre Antoine Lacassagne, Medical Oncology Department, Nice, France

Correction to: *Scientific Reports* 10.1038/s41598-020-60437-0, published online 27 February 2020

This Article contains a typographical error in the Introduction where the following is repeated twice:

“A single response profile, such as pseudo-progression, is observed under CPIs^7^. Among these typically-related response profiles under CPIs is hyperprogressive disease (HPD), which was defined as an unanticipated and paradoxical acceleration of the tumor growth^7,8^. The incidence of HPD is variable according to the way it is defined and ranges between 4 and 29%^7^. Though such acceleration of the tumor growth kinetic was also observed with other agents (chemotherapy^9^, tyrosine kinase inhibitors^10^), the intensity and the frequency of the phenomenon appears to be higher with checkpoint inhibitors used alone^7^.”

Additionally, Table 4 contains an error where the labels for ‘Risk Group’ and ‘Hyperprogressive Disease’ are swapped. The correct Table 4 appears below.

**Table 4:** .

Hyperprogressive disease	Total n (%)	Risk Group		Odds Ratio (CI 95%)	p
		Low risk	High risk		
No HPD	69 (86.25%)	66 (95.5%)	3 (4.5%)	1.0 referent	
HPD	11 (13.75%)	6 (54.5%)	5 (45.5%)	18.34 [3.38–99.58]	<0.001

